# Unraveling the genetic background of individuals with a clinical familial hypercholesterolemia phenotype

**DOI:** 10.1016/j.jlr.2023.100490

**Published:** 2023-12-18

**Authors:** Ana Margarida Medeiros, Ana Catarina Alves, Beatriz Miranda, Joana Rita Chora, Mafalda Bourbon, investigators of the Portuguese FH Study, Mafalda Bourbon, Quitéria Rato, Ana Catarina Alves, Ana Margarida Medeiros, Ana Catarina Gomes, Ana Cristina Ferreira, Ana Gaspar, Ana Margarida Marques, Ana Maria Garabal, Ana Paula Bogalho, Ana Rita Pereira, Anabela Raimundo, André Travessa, Andreia Lopes, António Afonso, António Furtado, António Guerra, António Monteiro, António Trindade, Armindo Ribeiro, Bernardo Dias Pereira, Bernardo Marques, Carla Laranjeira, Catarina Senra Moniz, Cecília Frutuoso, Cláudia Falcão Reis, Cláudia Rodrigues, Clementina Fernandes, Conceição Ferreira, Daniel Ferreira, Diogo Torres, Elisabete Martins, Elsa Gaspar, Fabiana Pimentel, Fernando Simões, Francisco Araújo, Francisco Silva, Goreti Lobarinhas, Graça Morais, Guida Gama, Guilherme Lourenço, Helena Mansilha, Helena Pereira, Heloísa Santos, Henedina Antunes, Inês Batista Gomes, Inês Colaço, Isabel Azevedo, Isabel Palma, João Anselmo, João Porto, João Ramos, João Sequeira Duarte, Jorge Pintado Alves, José Miguel Salgado, José Pereira de Moura, Leonor Sassetti, Lina Cardoso Ramos, Luísa Diogo Matos, Luísa Mota Vieira, Luísa Pires, Márcio de Moura, Margarida Bruges, Margarida Venâncio, Maria do Rosário Barroso, Maria João Virtuoso, Maria Luísa Gonçalves, Mário Martins Oliveira, Mendes Nunes, Miguel Costa, Miguel Mendes, Miguel Toscano Rico, Mónica Tavares, Natalina Miguel, Oana Moldovan, Olga Azevedo, Patrícia Lipari Pinto, Patrícia Pais, Patrícia Vasconcelos, Paula Garcia, Paula Martins, Pedro Marques da Silva, Piedade Lemos, Quitéria Rato, Raquel Coelho, Raquel Gouveia da Silva, Raquel Ribeiro, Rita Jotta de Oliveira, Roberto Pinto, Sandra Pereira, Sérgio Ferreira Cristina, Sílvia Sequeira, Susana Correia, Tânia Vassalo, Tiago Pack, Vânia Martins, Vera Frazão Vieira

**Affiliations:** 1 Unidade de I&D, Grupo de Investigação Cardiovascular, Departamento de Promoção da Saúde e Prevenção de Doenças Não Transmissíveis, Instituto Nacional de Saúde Doutor Ricardo Jorge, Lisboa, Portugal; 2 BioISI – Biosystems & Integrative Sciences Institute, Faculdade de Ciências, Universidade de Lisboa, Lisboa, Portugal

**Keywords:** familial hypercholesterolemia, FH-phenocopy genes, polygenic hypercholesterolemia, hyper-Lp(a)

## Abstract

Familial hypercholesterolemia (FH) is a common genetic disorder of lipid metabolism caused by pathogenic/likely pathogenic variants in *LDLR*, *APOB*, and *PCSK9* genes. Variants in FH-phenocopy genes (*LDLRAP1*, *APOE*, *LIPA*, *ABCG5*, and *ABCG8*), polygenic hypercholesterolemia, and hyperlipoprotein (a) [Lp(a)] can also mimic a clinical FH phenotype. We aim to present a new diagnostic tool to unravel the genetic background of clinical FH phenotype. Biochemical and genetic study was performed in 1,005 individuals with clinical diagnosis of FH, referred to the Portuguese FH Study. A next-generation sequencing panel, covering eight genes and eight SNPs to determine LDL-C polygenic risk score and *LPA* genetic score, was validated, and used in this study. FH was genetically confirmed in 417 index cases: 408 heterozygotes and 9 homozygotes. Cascade screening increased the identification to 1,000 FH individuals, including 11 homozygotes. FH-negative individuals (phenotype positive and genotype negative) have Lp(a) >50 mg/dl (30%), high polygenic risk score (16%), other monogenic lipid metabolism disorders (1%), and heterozygous pathogenic variants in FH-phenocopy genes (2%). Heterozygous variants of uncertain significance were identified in primary genes (12%) and phenocopy genes (7%). Overall, 42% of our cohort was genetically confirmed with FH. In the remaining individuals, other causes for high LDL-C were identified in 68%. Hyper-Lp(a) or polygenic hypercholesterolemia may be the cause of the clinical FH phenotype in almost half of FH-negative individuals. A small part has pathogenic variants in *ABCG5/ABCG8* in heterozygosity that can cause hypercholesterolemia and should be further investigated. This extended next-generation sequencing panel identifies individuals with FH and FH-phenocopies, allowing to personalize each person’s treatment according to the affected pathway.

Familial hypercholesterolemia (FH) is a common genetic disorder of lipid metabolism with a prevalence of 1:300 in the general population and even higher among patients with coronary artery disease (CAD) (1). An FH individual presents an increased risk of CAD because of lifelong exposure to severely elevated LDL-C (2).

Clinical diagnosis of FH is made following specific criteria as the Simon Broome Register Diagnostic Criteria or Dutch Lipid Clinic Network Diagnostic Criteria, both based on clinical features like LDL-C elevation, the presence of physical signs of cholesterol deposition, and family history of high LDL-C and/or premature CAD. However, FH genetic testing is the reference method to confirm a clinical suspicion and provide a definite diagnosis of FH (3).

There are three primary genes associated with FH: *LDLR*, *APOB*, and *PCSK9*. More than 90% of the variants reported in FH patients occur in the *LDLR* gene, 5–10% in the *APOB*, and less than 1% are identified in the *PCSK9* (4). Individuals with FH usually present a heterozygous pathogenic variant in one of these three genes but more rarely can have biallelic pathogenic variants in these genes presenting a more severe phenotype consistent with homozygous FH. Since both alleles can contribute to the phenotype, additively raising the LDL-C level, this inheritance pattern is described as autosomal semidominant (5).

Despite the technological advances in genetic diagnosis, FH is still underdiagnosed in most countries and, when a patient is identified, this is done late in life when they are already at risk of developing premature CAD (2). Based on the clinical criteria, only 40–70% have a pathogenic variant in one of the three FH primary genes (6, 7, 8, 9), so individuals who are phenotype positive and genotype negative (FH negative) may have another genetic cause contributing to their raised LDL-C levels.

Next-generation sequencing (NGS) enables the identification of variants in several genes at the same time, and for FH, the optimal panel recommended is the three FH-causing genes and five FH-phenocopy genes (*APOE*, *LDLRAP1*, *LIPA*, *ABCG5*, and *ABCG8*) (3), which are associated to other disorders that present a similar phenotype as FH. In fact, a rare deletion in *APOE* gene p.Leu167del (10) and also biallelic pathogenic variants in *LDLRAP1* (11), *LIPA* (12), *ABCG5*, or *ABCG8* (13) genes have all been reported in individuals with clinical FH phenotype with markedly elevated LDL-C levels.

A fraction of FH-negative cases may have a polygenic hypercholesterolemia, carrying a high burden of common LDL-C-raising alleles that, in complex interaction with environmental and lifestyle factors, leads to a clinical FH phenotype (14, 15).

Elevated lipoprotein (a) [Lp(a)] levels were observed in individuals with clinical FH phenotype, and no pathogenic variant was identified in the eight mentioned genes (16). Since the Lp(a) levels are known to be genetically determined and the LDL-C in the Lp(a) particle is known to contribute to the total LDL-C concentration, the phenotype of individuals with high Lp(a) can often mimic the clinical FH phenotype (17, 18). This can be in part explained by an increased frequency of common Lp(a)-raising alleles in *LPA* gene (rs3798220 and rs10455872) among these individuals (19). It has been described that these SNPs are associated with higher Lp(a) concentrations and explain about 50% of the cases with small apo(a) isoforms (18).

In the present work, we describe the implementation of a diagnostic NGS panel including the three FH primary genes and the five FH-phenocopy genes as well as common SNPs related to LDL-C and Lp(a) levels. These results together with Lp(a) biochemical levels allowed us to characterize the genetic background of 82% of our cohort.

## Materials and Methods

### Study design and index-case selection

The Portuguese FH Study is a research project, free of charge for all participants and health institutions, coordinated by the National Health Institute Dr Ricardo Jorge (INSA) (20). Since 1999, individuals with a clinical diagnosis of FH are being referred by several collaborating centers (including cardiologists, internists, endocrinologists, geneticists, pediatricians, and general practitioners). A clinical questionnaire is filled by the clinician for each index case, with pretreatment lipid levels, lipid-lowering treatment, physical examination, cardiovascular disease, and family history. Pretest and post-test genetic counseling is offered to all participants of the Portuguese FH Study by the referring clinician as recommended in the genetic report.

A complete biochemical profile is always determined at INSA at the time of referral, and participants are then evaluated. All index cases fulfilling Simon Broome criteria (21), possible or definite FH, are selected for molecular study. Cascade screening is always performed to the available family members in families identified with pathogenic, likely pathogenic variants, and variants of uncertain significance (VUSs).

Written informed consent is obtained for all participants before their inclusion in the study. The study protocol and database have been approved by the National Institute of Health Ethics Committee and the National Data Protection Commission.

### Biochemical profile

A fasting biochemical profile is performed for all participants at the time of referral to the study, including quantification of total cholesterol (TC), direct LDL-C, HDL-C, triglycerides, apolipoprotein A1, apolipoprotein B (apoB), and Lp(a). Lipids and lipoproteins are determined in a Cobas Integra 400 plus (Roche, Risch-Rotkreuz, Switzerland) by enzymatic colorimetric and immunoturbidimetric methods.

### Molecular study

#### FH panel

Between 2017 and 2021, the molecular study has been performed in 212 index cases by an extended targeted NGS panel with 57 lipid genes that are analyzed in three different genetic approaches: FH panel (8 genes), dyslipidemia panel (24 genes), and research panel (33 genes). In the scope of this work, only the FH panel has been analyzed. The FH panel includes three FH primary genes (*LDLR*, *APOB*, and *PCSK9*) and five FH-phenocopy genes (*LDLRAP1*, *APOE*, *LIPA*, *ABCG5*, and *ABCG8*). The targeted regions comprised the coding regions of each gene, the 50 bp flanking each intron and the 5′UTR region.

After genomic DNA extraction, samples were prepared according to SureSelect QXT Target Enrichment System protocol (Agilent Technologies). Libraries’ concentrations were determined using a Qubit® fluorometer (Thermo Fisher Scientific) and the Agilent® 2200 TapeStation (Agilent Technologies). Samples were pooled prior to sequencing to a final concentration of 4 nM and run in a NextSeq platform (Illumina) using a NextSeq 550 System generating 130M reads per run (2 × 75 base reads). The FASTQ files were analyzed using the SureCall software (Agilent Technologies). Output VCF files were analyzed using wANNOVAR software for identification of single nucleotide variants or deletions/insertions (22). Output BAM files were analyzed using DECoN-v1.0.2 software for copy-number variant identification (23). Overall, an average of 200–300 variants have been identified in each sample for the analysis of the panel of eight genes. The number of variants was narrowed down by excluding intronic variants located at more than 10 bp from the intron/exon boundaries and synonymous variants in *APOB* and *PCSK9*. Variants were also excluded if the allele frequency in the Genome Aggregation Database (gnomAD, version 2.1.1) (24) was greater than expected for the disorder (25): variants with a minor allele frequency above 1% in genes related with a dominant inheritance (*LDLR*, *APOB*, *PCSK9*, and *APOE*) or with a minor allele frequency above 5% in genes related with recessive inheritance (*LDLRAP1*, *LIPA*, *ABCG5*, and *ABCG8*). The identified rare variants were confirmed by Sanger sequencing and multiplex ligation-dependent probe amplification for copy-number variants in *LDLR* gene. Target Sanger sequencing or *LDLR* multiplex ligation-dependent probe amplification was also performed to all available family members for variant cosegregation analysis.

#### Polygenic risk scores

The NGS FH panel also captures SNPs related with two genetic risk scores associated with LDL-C (6-SNP LDL-C polygenic risk score [PRS]) and Lp(a) levels (*LPA* genetic score). In the present work, we present the genotyping results for 708 individuals with the 6-SNP LDL-C score and 202 individuals with the *LPA* genetic score.

The 6-SNP LDL-C PRS was calculated using the weighted sum of the risk alleles, that is, multiplying the number of LDL-C-raising risk alleles by the corresponding effect sizes of each SNP rs629301 (*CELSR2/SORT1*), rs1367117 (*APOB*), rs4299376 (*ABCG5/8*), rs6511720 (*LDLR*), rs7412 and rs429358 (*APOE*), as previously described (15). A high PRS was considered for an LDL-C score ≥0.76 (>75th percentile of the score distribution in the Portuguese population), and a low PRS was considered for an LDL-C score <0.51 (<50th percentile) (26).

The *LPA* genetic score results from the sum of the risk alleles of the two SNPs (C for rs3798220 and G for rs10455872) previously described (27). *LPA* risk score was considered if *LPA* genetic score ≥1, that is, if at least one *LPA* risk allele is present.

#### Resequencing of phenotype-positive and genotype-negative (FH-negative) individuals

In 2017, 141 index cases previously studied by Sanger sequencing (26, 28) where a pathogenic/likely pathogenic variant was not found in one of the three primary FH genes, were investigated for seeking other causes of their severe phenotype. This was performed using a diagnostic and research NGS panel with 112 genes involved in lipid metabolism, sequenced through a collaborative project at the European Molecular Biology Laboratory (EMBL). The targeted regions included coding regions plus 50 bp flanking each intron and the 5′UTR region. Genomic DNA samples were prepared according to HaloPlex™ Target Enrichment System Kit (Agilent Technologies) and run on an Hiseq (Illumina) located at EMBL. In this work, we will only report the variants identified in the eight gene FH panel. A sample of 143 Portuguese normolipidemic individuals (LDL-C <115 mg/dl) from the e_COR study was also sequenced using this panel (29).

### Variant classification

The American College of Medical Genetics and Genomics (ACMG) recommends five variant classification categories: pathogenic, likely pathogenic, VUS, likely benign, and benign. *LDLR* variants were classified according to the ACMG guidelines (25) and the Clinical Genome Resource (ClinGen) FH Variant Curation Expert Panel consensus guidelines for *LDLR* variant classification (30). *APOB* and *PCSK9* variants were classified according to the ACMG guidelines (25), using specific published adaptations for these genes (31). Variants in the remaining genes of the panel were classified according to the ACMG general guidelines (25).

Variants in FH-phenocopy genes were classified for their specific phenocopy: *ABCG5* and *ABCG8* for sitosterolemia, *APOE* for dysbetalipoproteinemia, and *LIPA* for lysosomal acid lipase deficiency.

The identified variants were checked with Mutalyzer 3.0.4, (https://mutalyzer.nl/) as recommended by the Human Genome Variation Society. The sequence references used for analysis were NM_000527.5 for *LDLR*, NM_000384.3 for *APOB*, NM_174936.4 for *PCSK9*, NM_000041.4 for *APOE*, NM_015627.3 for *LDLRAP1*, NM_022436.3 for *ABCG5*, NM_022437.3 for *ABCG8*, and NM_000235.4 for *LIPA*.

### Cosegregation studies

For cosegregation analysis, affected individuals were considered as such when untreated TC or LDL-C levels were above the 75th percentile adjusted for age and sex for the Portuguese population (32). Whenever untreated TC or LDL-C values for individuals under statin treatment were not available, general 0.8 and 0.7 correction factors were applied, corresponding to a conserved estimation of 20% TC and 30% LDL-C reduction on treatment, respectively (30). Family members were considered unaffected when they had an untreated TC and LDL-C below the 50th percentile adjusted for age and sex (30).

### Statistical analysis

Statistical analysis was performed using SPSS software (IBM SPSS Statistics for Windows, version 27.0; IBM Corp, Armonk, NY). Frequencies of qualitative variables were compared using the Chi-square test. Mean values of quantitative variables were compared with the Student’s *t*-test for independent data, and median values were compared with nonparametric Mann-Whitney median test. *P* value <0.05 was considered statistically significant.

## Results

**Supplemental Figure S1:**
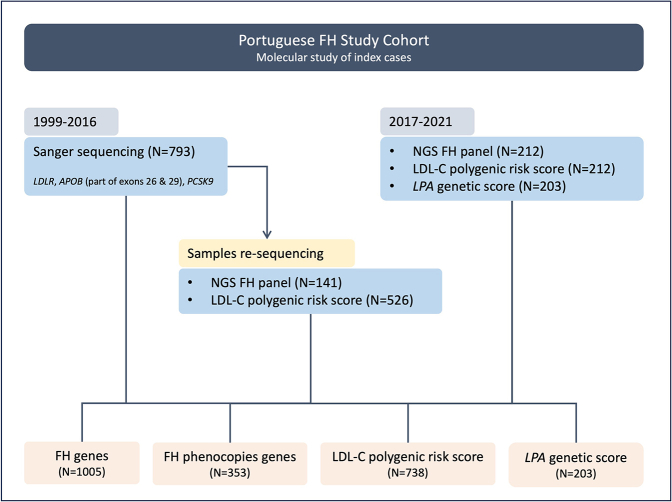

Between 1999 and 2021, individuals with clinical FH were studied using different methodologies described in supplemental Fig. S1.

### NGS sequencing

#### FH panel

The NGS panel was first validated with 16 samples with known variants in seven of the eight genes (supplemental Table S1), and all variants were detected by this methodology (100% of sensitivity and specificity).

Between 2017 and 2021, the genetic diagnosis of FH was performed in 212 index cases using the eight gene FH panel. A total of 69 index cases, 34 children and 35 adults, were identified with heterozygous FH (32.9%) carrying pathogenic or likely pathogenic variants in the three primary genes. One child was also identified as compound heterozygous carrying two variants (one pathogenic and one likely pathogenic) in the *LDLR* gene (Table 1). Most of the FH-positive individuals had *LDLR* variants (N = 65; 93%), pathogenic or likely pathogenic, but pathogenic variants in *APOB* (N = 3; 4.3%) or *PCSK9* (N = 1; 1.4%) were also identified. One of the three index cases carrying a pathogenic *APOB* variant was also identified with a VUS in the *LDLR* gene.

**Table 1:** Clinical and genetic characteristics of the homozygous FH of the Portuguese FH Study

Demographic			Clinical data		Lipid profile (mg/dl)						Variant			
Gender	Age at referral (years)	Present age in years (alive or deceased)	CHD (age in years)	Tendon xanthoma		TC	LDL-C	HDL-C	TG	Treatment	Gene	Complementary DNA	Protein	Protein activity
F	55	77 (?)	No	No	*Pre*	—	—	—	—	Statin	*LDLR*^a^	**c.[1285G>C];[1285G>C]**	**p.[(Val429Leu)];[(Val429Leu)]**	15–30% LDLR activity
					*On*	299	232	47	99					
F	25	41 (Alive)	No	No	*Pre*	596	512	65	96	Statin + ezetimibe	*LDLR*^a^	**c.[1291G>A];[1291G>A]**	**p.[(Ala431Thr)];[(Ala431Thr)]**	∼20% LDLR activity
					*On*	370	299	54	84					
F	21	34 (Alive)	No	No	*Pre*	345	210	51	—	Statin	*LDLR*^a^	**c.[1216C>T];[c.1216C>T]**	**p.[(Arg406Trp)];[(Arg406Trp)]**	60–65% LDLR activity
					*On*	258	186	50	79					
F	36	57 (Alive)	No	No	*Pre*	490	435	42	64	Rosuvastatin + ezetimibe	*LDLR*^b^	c.[313+6C>T];	p.[Leu64_Pro105delinsSer];	?
					*On*	355	280	51	219			**c.[1291G>A]**	**p.[(Ala431Thr)]**	∼20% LDLR activity
M	29	45 (Deceased)	MI (23)	No	*Pre*	561	515	—	—	Statin + ezetimibe + LDL apheresis	*LDLR*^b^	**c.[631C>G;1816G>T];**	**p.[(His211Asp);(Ala606Ser)];**	Normal cell surface LDLR (100%), ∼50% binding and uptake
					*On*	346	298	29	96			**c.[1178delA]**	**p.[(Lys393Argfs∗20)]**	<10% LDLR activity
F	46	57 (?)	No	No	*Pre*	600	**—**	**—**	**—**	Statin + ezetimibe	*LDLR*^b^	**c.[1633G>T];**	**p.[(Gly545Trp)];**	∼10% LDLR activity
					*On*	185	108	63	36			**c.[1775G>A]**	**p.[(Gly592Glu)]**	51% expression, ∼40% binding and uptake
F	61	71 (Alive)	Angina (50)	No	*Pre*	587	401	69	67	Statin + ezetimibe + LDL apheresis	*LDLR*^b^	**c.[(1060+1_1061-1)_(1845+1_1846-1)del];**	**p.[?];**	<10% LDLR activity
					*On*	389	293	65	122			**c.[1216C>T]**	**p.[(Arg406Trp)]**	60–65% LDLR activity
F	10	17 (Alive)	No	No	*Pre*	656	594	45	70	Statin	*LDLR*^b^	**c.[670G>A];**	**p.[(Asp224Asn)];**	<10% LDLR activity
					*On*	470	397	38	70			**c.[1291G>A]**	**p.[(Ala431Thr)]**	∼20% LDLR activity
F	15	19 (Alive)	No	No	*Pre*	423	317	41	323	Atorvastatin	*LDLR*^b^	**c.[1060+1G>A];**	**p.[(Gly293_Glu332del)];**	?
					*On*	398	296	44	339			**c.[1585G>C]**	**p.[(Gly529Arg)]**	∼69% LDLR activity
F	11	22 (Alive)	No	No	*Pre*	316	234	50	58	Statin	*PCSK9*^b^	**c.[185C>A];**	**p.[(Ala62Asp)];**	46% expression and ∼35% uptake
					*On*	139	88	42	55			c.[1399C>G]	p.[(Pro467Ala)]	56% expression and ∼35% uptake
F	21	22 (Alive)	No	No	*Pre*	465	356	78	149	No	*LDLR*	**c.1291G>A;**	**p.(Ala431Thr)**	∼20% LDLR activity
					*On*	—	—	—	—		*APOB*	**c.10580G>A**	**p.(Arg3527Gln)**	∼40% LDL binding and uptake

?, unknown; CHD, coronary heart disease; F, female; M, male; MI, myocardial infarction; on, on treatment value; pre, pretreatment value; TG, triglyceride.

a True homozygous.

b Compound homozygous; pathogenic or likely pathogenic variants (ACMG classification) are in bold, the remaining are VUS.

In the remaining 142 index cases (67%), a pathogenic variant or likely pathogenic variant was not identified in the three primary genes. However, six presented a VUS in the *LDLR* gene, and 27 (19%) presented a VUS in *APOB* and/or *PCSK9* genes. In 20% of the FH-negative individuals (N = 28), heterozygous variants were identified in the five FH-phenocopy genes (supplemental Table S2). A high polygenic LDL-C risk score was identified in 57 individuals (40%) and an *LPA* risk score ≥1 in 35 individuals (25%) of the FH-negative cohort. This will be presented in detail when describing the whole cohort.

#### Resequencing of phenotype-positive and genotype-negative (FH-negative) individuals

Two individuals were identified with a likely pathogenic variant in *APOB* gene (c.11477C>T/p.(Thr3826Met)), outside the region previously sequenced by Sanger sequencing. Another four individuals were identified with pathogenic variants in *LDLR* gene, which were missed by Sanger sequencing technique. No other pathogenic variants or likely pathogenic variants were identified in the remaining genes of the FH panel, but 14% (N = 19) of these FH-negative individuals presented a VUS in *APOB* and/or *PCSK9* genes. In 17% (N = 23) of the individuals, a heterozygous variant in the five FH phenocopies was identified. These results were added in the analysis of the whole cohort below.

### Update on the Portuguese FH Study

Between 1999 and 2021, a total of 1,005 index cases with clinical diagnosis of FH following Simon Broome criteria have been referred to the Portuguese FH Study. Until 2021, we have confirmed genetically 417 FH index cases (FH positive): 408 with heterozygous FH and 9 with homozygous FH (three true homozygous and six compound heterozygous). Heterozygous FH individuals have most frequently pathogenic variants or likely pathogenic variants in *LDLR* (N = 381; 93%) and less frequently in *APOB* (N = 22; 5%) and *PCSK9* (N = 5; 1%) genes. In the homozygous FH group, eight of nine have variants in the *LDLR* gene. All variants were classified using ACMG guidelines as described in the *Methods* section, and evidence codes applied to each variant are presented in supplemental Table S3. VUS in the FH primary genes were identified in 73 individuals: *LDLR* (N = 24), *APOB* (N = 45), and *PCSK9* (N = 4).

**Fig. 1:**
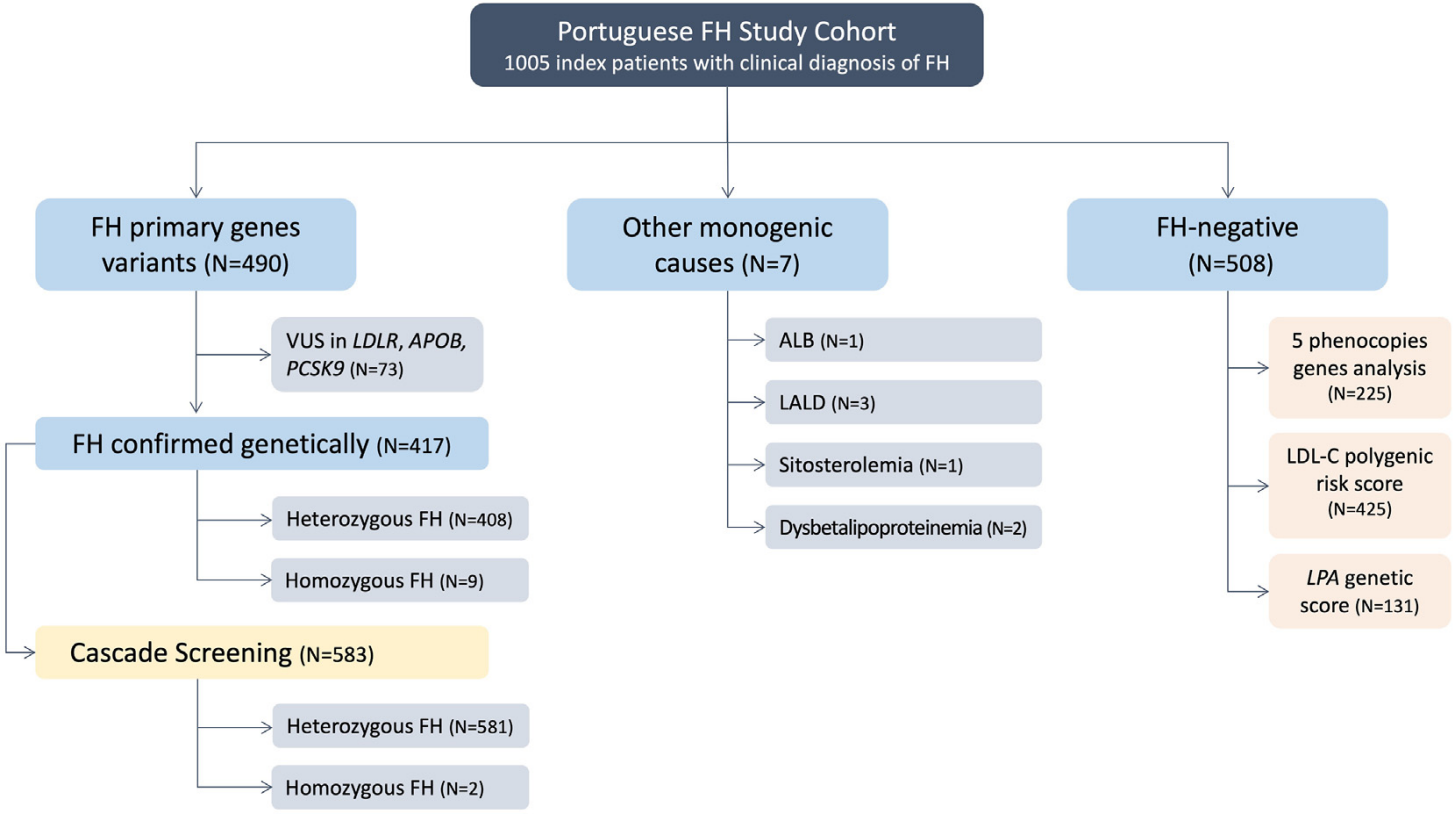
Diagram of molecular study in the FH cohort. About 1,005 individuals with clinical diagnosis of FH were selected for molecular study: 417 have FH confirmed genetically, 73 carry VUS in FH primary genes, and 7 have other monogenic causes. In the group of FH-negative individuals: 226 were analyzed for 5 FH-phenocopy genes, 425 for LDL-C PRS, and 131 for LPA genetic score.

Cascade screening performed in these 417 FH families allowed the identification of further 583 FH individuals: 581 with heterozygous FH and 2 with biallelic pathogenic variants in FH genes (one *LDLR* compound heterozygous and one *LDLR-APOB* double heterozygote). So, in total, 1,000 individuals received a definite diagnosis of FH: 989 heterozygous FH and 11 homozygous FH (Fig. 1). The demographic, clinical, and genetic characteristics of the homozygous individuals are presented in Table 1.

Other monogenic causes, as lysosomal acid lipase deficiency, sitosterolemia, congenital analbuminemia, dysbetalipoproteinemia, have been identified in seven index cases of our cohort, six of them reported previously (12, 26) (Fig. 1).

#### Clinical characteristics of FH positive *versus* FH negative

All individuals with heterozygous pathogenic variants or likely pathogenic variants in the FH primary genes were included in the FH-positive group. Individuals carrying *LDLR* VUS and other monogenic causes were excluded from this analysis.

The remaining individuals in the cohort (58%) where no pathogenic variant or likely pathogenic variant were identified in the primary FH genes were classified as FH negative. Individuals with VUS in *APOB* and *PCSK9* were also included in this group.

The demographic and clinical characteristics, as well as the lipid profile, of the FH-positive and FH-negative individuals were compared and are presented in supplemental Table S4.

#### FH negative: identification of variants in FH-phenocopy genes

The five phenocopy genes (*LDLRAP1*, *APOE*, *LIPA*, *ABCG5*, and *ABCG8*) were analyzed in 225 individuals (Fig. 1). As a result, we found 35 different variants in 52 of the 225 FH-negative individuals analyzed (23%) (supplemental Table S2). Among these, 12 individuals (5.3%) carried one heterozygous pathogenic (frameshift or stop) variant in *ABCG5* (N = 5; 2.2%), *ABCG8* (N = 6; 2.7%), or *APOE* (N = 1; 0.4%). One of these carried both a heterozygous pathogenic variant and a heterozygous VUS in *ABCG8*. Thirty-six individuals were identified with one heterozygous VUS in *ABCG5* (N = 18; 8.0%), *ABCG8* (N = 14; 6.2%), *APOE* (N = 3; 1.3%), or *LIPA* (N = 1; 0.4%). Four individuals carried two heterozygous VUS in *ABCG5* (N = 2; 0.9%) or in both genes (N = 2; 0.9%). GC-MS was performed in our institute for the five individuals with biallelic variants in *ABCG5/ABCG8* genes, but high levels of beta-sitosterol were not detected in any of them (supplemental Table S5).

We have not identified rare variants in *LDLARP1* gene in FH-negative individuals.

LDL-C mean levels in individuals with heterozygous variants in FH-phenocopy genes were significantly lower when compared with FH-positive individuals (children’s cohort: 168.6 ± 21.6 mg/dl *vs.* 217.1 ± 49.7 mg/dl, *P* = 0.001; adult’s cohort: 215.1 ± 67.5 mg/dl *vs.* 268.2 ± 96.6 mg/dl, *P* = 0.009). There was no statistically significant difference between LDL-C mean levels of individuals carrying heterozygous variants in FH-phenocopy genes when compared with FH-negative individuals without variants in FH phenocopies.

#### FH negative: identification of other causes (hyper-Lp(a) and PRS)

Hyper-Lp(a) was considered for Lp(a) levels >50 mg/dl and was significantly more prevalent in FH-negative individuals when compared with FH-positive individuals of our cohort (38% *vs.* 32%, *P* = 0.035). The frequency of hyper-Lp(a) was also significantly higher in the pediatric FH-negative group when compared with FH-positive group (41.9% *vs.* 25.4%, *P* < 0.001) (supplemental Table S4). In the adult’s cohort, there was no statistically significant difference in the prevalence of hyper-Lp(a) between FH-negative and FH-positive individuals (supplemental Table S4). LDL-C mean levels of FH-negative individuals with hyper-Lp(a) were significantly lower than LDL-C mean levels of heterozygous FH individuals (children’s cohort: 165.4 ± 37.7 mg/dl *vs.* 217.1 ± 49.7 mg/dl, *P* < 0.001; adult’s cohort: 204.6 ± 61.2 mg/dl *vs.* 268.2 ± 96.6 mg/dl, *P* < 0.001). There was no statistically significant difference between LDL-C mean levels of FH-negative individuals with hyper-Lp(a) when compared with FH-negative individuals with normal Lp(a) levels (children’s cohort: 165.4 ± 37.7 mg/dl *vs.* 160.0 ± 39.5 mg/dl, *P* = 0.541; adult’s cohort: 204.6 ± 61.2 mg/dl *vs.* 208.0 ± 66.2 mg/dl, *P* = 0.435).

One or two *LPA* risk alleles were detected in 84 of the 131 FH-negative individuals genotyped (64%). There was no statistically significant difference between the Lp(a) mean levels of FH-negative individuals with an *LPA* risk score of ≥1 and FH-negative individuals with *LPA* risk score of 0 (45.6 ± 44.1 mg/dl *vs.* 38.4 ± 43.3 mg/dl, *P* = 0.269). However, 61% of the individuals with hyper-Lp(a) present at least one *LPA* risk allele.

A high polygenic LDL-C risk score (>75th percentile) was identified in 190 of the 425 FH-negative individuals genotyped (44.7%). The mean PRS was significantly higher in FH-negative individuals when compared with FH-positive individuals both in the pediatric group (0.74 ± 0.17 *vs.* 0.67 ± 0.20, *P* = 0.007) and in the adult’s group (0.71 ± 0.19 *vs.* 0.67 ± 0.20, *P* = 0.044) (supplemental Table S4). LDL-C mean levels of FH-negative individuals with high PRS were significantly lower than LDL-C mean levels of heterozygous FH individuals (children’s cohort: 162.5 ± 36.5 mg/dl *vs.* 217.1 ± 49.7 mg/dl, *P* < 0.001; adult’s cohort: 204.3 ± 66.5 mg/dl *vs.* 268.2 ± 96.6 mg/dl, *P* <0.001). There was no statistically significant difference between LDL-C mean levels of FH-negative individuals with high PRS when compared with FH-negative individuals with PRS below the 75th percentile.

#### Hyper-Lp(a) associated with CAD

The frequency of CAD and premature CAD was significantly higher in FH-negative individuals with hyper-Lp(a) when compared with FH-negative individuals with normal Lp(a) levels (CAD: 27.3% *vs.* 16.7%, *P* = 0.038; pCAD: 24.5% *vs.* 14.1%, *P* = 0.029). In the FH-positive cohort, this frequency was also significantly higher in individuals with hyper-Lp(a) when compared with individuals with normal Lp(a) levels (CAD: 31.5% *vs.* 13.6%, *P* = 0.005; pCAD: 27.4% *vs.* 9.3%, *P* = 0.002).

### Genetic background of individuals with clinical FH phenotype

**Fig. 2:**
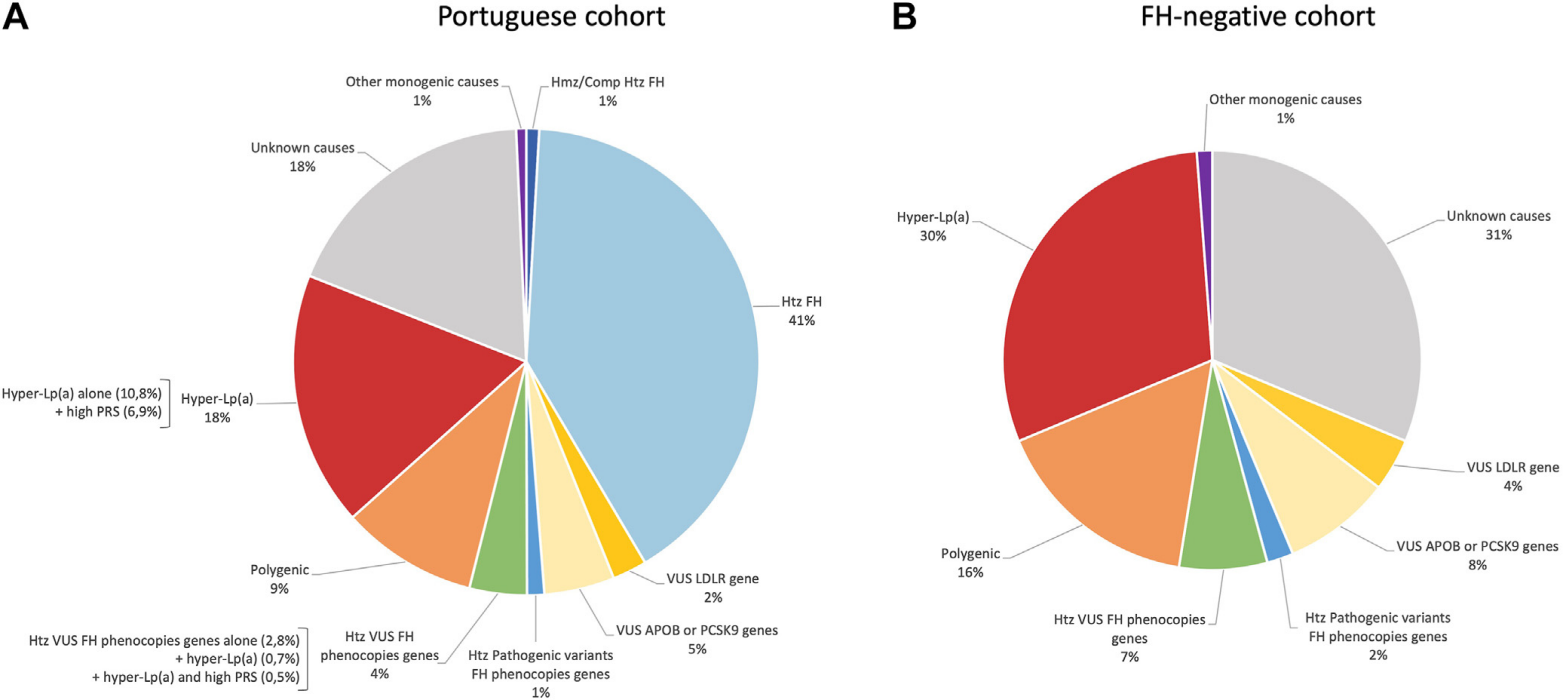
A: Genetic background of individuals with clinical FH phenotype from the Portuguese cohort. B: Genetic background of FH-negative individuals. Comp Htz, compound heterozygous; Hmz, homozygous; Htz, heterozygous.

Overall, in the Portuguese FH Study cohort, FH was genetically confirmed in 42% (N = 417) of all index cases with clinical diagnosis of FH. This includes individuals with homozygous FH that showed the same frequency as other monogenic lipid metabolism disorders (1%) in all index cases (Fig. 2A). In the remaining 588 individuals (58%), several causes have been identified, beyond other monogenic lipid disorders: 30% (N = 177) of the FH-negative individuals present hyper-Lp(a), 16% (N = 95) present a high LDL-C PRS, and 2% (N = 12) have one pathogenic variant in the *ABCG5*, *ABCG8*, or *APOE* genes. In addition, 12% (N = 73) carry heterozygous VUS in FH primary genes, and 7% (N = 40) carry heterozygous VUS in FH-phenocopy genes (Fig. 2B).

LDL-C mean levels of the FH individuals present in each subgroup are presented in supplemental Table S6. Heterozygous FH-positive individuals (pediatric and adult cohort) present LDL-C mean levels statistically higher when compared with FH-negative individuals of each subgroup. There was no statistically significant difference between LDL-C mean levels in each subgroup of FH-negative individuals.

## Discussion

In the present work, we describe the implementation of a diagnostic NGS panel that allows not only the genetic confirmation of FH but also the identification of variants in FH-phenocopy genes, polygenic hypercholesterolemia, and *LPA* genetic score, as recommended in the consensus paper on FH diagnosis (3).

The aim of this work was to unravel the genetic background in individuals with clinical diagnosis of FH. Our approach was based on NGS technology, and the designed NGS panel contains three FH primary genes (*LDLR*, *APOB*, and *PCSK9*) and five FH-phenocopy genes (*LDLRAP1*, *APOE*, *LIPA*, *ABCG5*, and *ABCG8*). This panel has been validated and is in use in our laboratory since 2017 to perform the genetic characterization of individuals with clinical diagnosis of FH referred to the Portuguese FH Study. During 2017 to 2021, this diagnostic approach identified pathogenic/likely pathogenic FH-causing variants in 33% of the clinically diagnosed FH individuals, a slightly lower detection rate compared with the previously observed in our cohort (26, 28). This lower detection rate may be related to the fact that the new guidelines for variant classification in the *LDLR* gene, recently published by the FH Variant Curation Expert Panel, are more conservative than the general ACMG guidelines (30). However, the overall diagnostic positive yield between 1999 and 2021 in our cohort was 41%, which is within the range observed in many other countries like Spain (40%) (9), Canada (50%) (8) and Brazil (50.4%) (7).

After cascade screening, by the end of December 2021, we have genetically identified a total of 1,000 individuals with FH: 989 with heterozygous FH and 11 with homozygous FH (three true homozygous, seven compound heterozygous, and one *LDLR-APOB* double heterozygote). As expected, the identified FH individuals have most frequently pathogenic or likely pathogenic variants in *LDLR* and less frequently in *APOB* and *PCSK9* genes (4). In a small part of the cohort (7%), variants of unknown significance were identified in *LDLR*, *APOB*, and *PCSK9* genes. These variants need to be functionally characterized to add evidence to upgrade or downgrade their classification (33, 34, 35). Individuals with VUS in *APOB* and *PCSK9* genes were considered in the FH-negative group, as these genes are highly polymorphic, and most VUS in these genes do not affect LDLR activity and therefore are not the cause of FH. On the other hand, about 50% of the VUS in *LDLR* are reclassified as pathogenic or likely pathogenic when more evidence is available like functional studies, case data, or cosegregation analysis (36) and, for this reason, these individuals were not considered in either the FH-positive groups or FH-negative groups for statistical analysis.

Other monogenic lipid metabolism disorders showed the same frequency as homozygous FH (1%), as previously reported (26).

As in other countries (7, 8) more than half of our clinical FH individuals cannot be confirmed genetically. FH-negative individuals present lower mean levels of TC, LDL-C, ApoB, and ApoB/apolipoprotein A1 ratio when compared with FH individuals genetically confirmed (supplemental Table S4), as reported before in our cohort (26, 37). These differences were observed both in the pediatric and adults’ cohorts. However, although FH-negative individuals have a milder phenotype, they still present clinical criteria of FH, so we sought to identify other genetic causes potentially related to the clinical FH phenotype in those individuals.

FH-phenocopy genes (*LDLRAP1*, *APOE*, *LIPA*, *ABCG5*, and *ABCG8*) were analyzed in a fraction of our FH-negative cohort (N = 225) to identify putative pathogenic variants that can contribute to their high LDL-C levels. Twelve individuals (∼1% of the entire cohort) were identified with one heterozygous loss-of-function variant (frameshift or nonsense) in *ABCG5*, *ABCG8*, or *APOE* genes. Almost all *ABCG5/ABCG8* loss-of-function variants identified in our cohort have been reported before in patients with sitosterolemia providing evidence to be classified as pathogenic or likely pathogenic (supplemental Table S2) (38, 39, 40, 41). Some of the *ABCG5* missense variants identified have been previously associated with non-HDL-C levels and with a greater risk of CAD in heterozygous carriers (42).

In our cohort, carriers of *ABCG5/ABCG8* variants presented lower LDL-C levels when compared with individuals with pathogenic variants in FH primary genes as observed in other cohorts (43, 44). Although, in some families, *ABCG5/ABCG8* missense variants did not shown complete segregation with high LDL-C levels, our data suggest that the presence of rare variants in FH-phenocopy genes may have an impact in LDL-C levels of these individuals, not as cause of FH but leading to a hypercholesterolemic phenotype. In fact, studies performed widely have reported pathogenic *ABCG5/ABCG8* variants to be associated with hypercholesterolemia (43, 45). Sitosterol levels have been reported previously to be increased in FH-negative individuals compared with normocholesterolemic controls and to be mildly elevated in heterozygous carriers of *ABCG5/ABCG8* variants (45, 46). One limitation of our study is that we have not determined beta-sitosterol levels for all *ABCG5/ABCG8* carriers, as for some of the index cases, it was not possible to collect new samples after the genetic diagnosis. For five individuals carrying two heterozygous variants in *ABCG5/ABCG8* genes, beta-sitosterol levels were quantified (supplemental Table S5) and, although some may be slightly elevated, we do not have beta-sitosterol levels from our normolipidemic population to compare. A discussion whether sitosterolemia should be considered codominant, as hypobetalipoproteinemia, should happen in the near future since heterozygous subjects present a phenotype of high LDL-C and raised beta-sitosterol.

A rare pathogenic *APOE* variant c.636_645del/p.(Val213Trpfs∗35) showed complete segregation with LDL-C levels (122.8 ± 18.1 mg/dl), and normal triglycerides, which may be the cause of hypercholesterolemia in this family. Variants in *APOE* gene are usually associated with dysbetalipoproteinemia; however, a rare *APOE* deletion have been reported to be associated to FH (10). Whether *APOE* variants are the cause of FH or dysbetalipoproteinemia remains to be discussed.

Hyper-Lp(a) was observed in 36% of the index cases analyzed in our cohort, similar to the prevalence reported before in a Copenhagen study where high Lp(a) levels have been identified in a quarter of the individuals with clinical diagnosis of FH (17). Although mean Lp(a) values were similar between FH-positive individuals and those without a pathogenic FH-causing variant, the frequency of hyper-Lp(a) was statistically significantly higher (*P* = 0.035) in FH-negative individuals (38%) when compared with FH-positive individuals (32%). In the adult’s cohort, there was no difference between FH-negative and FH-positive individuals, but in the pediatric cohort, the prevalence of hyper-Lp(a) was also statistically significantly higher (*P* < 0.001) in FH-negative individuals (41.9% *vs.* 25.4%). A recent study in a large cohort of children with suspected FH showed similar results (16). Our findings suggest that, in our cohort, hyper-Lp(a) may be the cause of hypercholesteremia in a larger number of FH-negative individuals.

In our study, *LPA* risk alleles (1 or 2) were present in more than half of the FH-negative individuals genotyped for *LPA* genetic score, but as expected, not all individuals with *LPA* risk alleles had elevated Lp(a). In fact, although these two SNPs are frequently associated with higher Lp(a) values, it has been described that approximately 50% of individuals with high Lp(a) values will remain undetected when only these two SNPs are genotyped, as observed in our cohort (47, 48). It is therefore important to measure Lp(a) in individuals with clinical diagnosis of FH to clarify the cause of their phenotype and to define adequate therapeutic measurements. Although we do not have specific treatments for hyper-Lp(a) now, it is important to identify these individuals and discuss what is the best approach to decrease their cardiovascular risk until new drugs are available.

We confirmed in our cohort that hyper-Lp(a) associates with a greater cardiovascular risk; we showed that CAD was statistically significantly more prevalent in adults with hyper-Lp(a) when compared with adults with normal Lp(a) levels (*P* < 0.001). If we stratify the results based on the presence or the absence of pathogenic variants in FH primary genes, the prevalence of CAD remains statistically significantly higher in individuals with hyper-Lp(a). This means that elevated Lp(a) could be an important risk modifier to take in consideration, worsening the risk of premature CAD in FH patients (19).

Along with high levels of Lp(a), FH-negative individuals also carry a high burden of common LDL-C-raising alleles, which confers a hypercholesteremia of polygenic origin rather than monogenic. Almost half (44.7%) of the FH-negative individuals genotyped for the 6-SNP score have a high PRS. In our study, a statistically significantly higher mean PRS was also observed in FH-negative individuals when compared with FH-positive individuals in both the pediatric and adult’s cohort. Similar results have been observed in other cohorts using this 6-SNP LDL-C score (49).

Overall, FH has been genetically confirmed in 42% (including 1% with homozygous FH) of the index cases with clinical diagnosis of FH referred to the Portuguese FH Study. A small part of the index cases was identified with VUS in the *LDLR* gene (2%) and *APOB* or *PCSK9* (5%), which need further studies, as functional studies, to upgrade their classification to likely pathogenic. In the remaining index cases, FH was not genetically confirmed, but other causes for high LDL-C have been identified as hyper-Lp(a), polygenic hypercholesterolemia, or variants in FH-phenocopy genes that should be further investigated (Fig. 2). The effect of these alternative causes of high LDL-C values may not be as large as pathogenic and likely pathogenic variants in FH genes but do confer high levels of LDL-C and will require different types of disease management, specific therapies, and may have implication on the cardiovascular risk stratification and cascade screening of relatives (3, 50).

On the other hand, some of these phenotype-positive and genotype-negative individuals may present variants in other genes that are involved in other metabolic pathways, as reported before (51, 52). Since the FH panel (eight genes) is part of an extended NGS covering 57 lipid genes related with hypertriglyceridemia and other dyslipidemias, it has provided a lot of genetic information on those genes, which are being analyzed and will be presented in a further work to better understand the genetic background of FH-negative patients.

Resequencing of FH-negative individuals by an NGS panel revealed that 3% had a pathogenic variant in *LDLR*, which were missed by Sanger sequencing methodology. This has also been reported by other groups that resequenced FH-negative cohorts and resulted most probably from human errors (52). *APOB* variants were also identified with the NGS panel, but these were outside the region studied by the previous methodology. This indicates that NGS technology has a greater sensitivity and includes all coding sequences of FH primary genes. So, resequencing is recommended, when possible, especially in FH-negative cohorts with subjects with a clear clinical FH phenotype.

The use of an extended FH NGS panel, as the one presented in this work, is important to identify the etiology of the hypercholesterolemia, specially at an early age, and therefore personalize each person’s treatment for a better prognosis (53).

## Data availability

The data are contained within the article and supplemental data file. The original data and dataset analyzed are available from the corresponding author upon reasonable request.

## Supplemental data

This article contains supplemental data (8, 10, 25, 30, 31, 38, 39, 40, 41, 42, 43).

## Conflict of interest

The authors declare that they have no conflicts of interest with the contents of this article.

