# Supplementary material for: Unraveling the genetic background of individuals with a clinical familial hypercholesterolemia phenotype
Source: J Lipid Res. 2023 Dec 18;65(2):100490. doi: 10.1016/j.jlr.2023.100490 (PMC10832474; doi:10.1016/j.jlr.2023.100490)
Supplement: Supplemental data [file mmc1.docx]

**Supplemental Data**

**Unravelling the genetic background of individuals with a clinical Familial Hypercholesterolemia phenotype**

Ana Margarida Medeiros, MSc^1,2^; Ana Catarina Alves, PhD^1,2^; Beatriz Miranda, MSc^1,2^; Joana Rita Chora, PhD^1,2^; Mafalda Bourbon, PhD^1,2^; on behalf of the investigators of the Portuguese FH Study

^1^Unidade de I&D, Grupo de Investigação Cardiovascular, Departamento de Promoção da Saúde e Prevenção de Doenças Não Transmissíveis, Instituto Nacional de Saúde Doutor Ricardo Jorge, Lisboa, Portugal;

^2^BioISI – Biosystems & Integrative Sciences Institute, Faculdade de Ciências, Universidade de Lisboa, Lisboa, Portugal

**Supplemental Table S1.** Samples with known variants used in the validation of the FH panel.

| **Sample** | **Gene** | **Variant** | **ClinVar ID** |
| --- | --- | --- | --- |
| **1** | *LIPA* | c.894G>A/p.Ser275_Gln298del | 203361 |
| **2** | *APOB* | c.11477C>T/p.(Thr3826Met) | 237735 |
| **3** | *LDLR* | c.[(?-1)_(190+1_191-1)del;(1060+1_1061-1)_(1845+1_1846-1)del] (Pr_EX2del + EX8_12del) | 250921 |
| **4** | *ABCG5* | c.[1890del];[1890del] / p.[(Phe630Leufs*8)];[(Phe630Leufs*8)] | 1684420 |
| **5** | *LIPA* | c.[894G>A];[894G>A] / p.[Ser275_Gln298del];[Ser275_Gln298del] | 203361 |
| **6** | *APOB* | c.13480_13482del/p.(Gln4494del) | 265896 |
| **7** | *LIPA* | c.[894G>A];[894G>A] / p.[Ser275_Gln298del];[Ser275_Gln298del] | 203361 |
| **8** | *LIPA* | c.894G>A/p.Ser275_Gln298del | 203361 |
| **9** | *LDLR* | c.1291G>A/p.(Ala431Thr) | 3695 |
| **10** | *LDLR* | c.1618_1620del/p.(Ala540del) | 251937 |
| **11** | *PCSK9* | c.[185C>A];[1399C>G] / p.[(Ala62Asp)];[(Pro467Ala)] | 265918; 265944 |
| **12** | *LDLR* | c.[1061-?_1845+?del];[1216C>T] / p.[(?)];[(Arg406Trp)] | 265901; 226351 |
| **13** | *LDLR* | c.618_638del/p.(Gly207_Ser213del) | 265899 |
| **14** | *LDLR* | c.77_78del/p.(Arg26Metfs*25) | 251008 |
| **15** | *APOB and ABCG8* | APOB: c.11477C>T/p.(Thr3826Met)  ABCG8: c.[1974C>G];[1974C>G] / p.[(Tyr658*)];[(Tyr658*)] | 237735  4969 |
| **16** | *APOE* | c.487C>T/p.(Arg163Cys) | 17851 |

**Supplemental Table S2**. Genetic variants of interest identified in FH phenocopy genes in FH-negative individuals of our cohort.

| **Genes/Variants** | **ClinVar ID** | **Nr index**  **(Htz carriers)** | **% PopMAF**  **(GnomAD v2.1.1)** | **Co-segregation** | **Allele frequency in normolipidemic^*^** | **Described previously** | **ACMG classification**^#^ |
| --- | --- | --- | --- | --- | --- | --- | --- |
| ***ABCG5*** |  | **N=30** |  |  |  |  |  |
| c.293C>G/p.(Ala98Gly) | 289815 | 4 | 1.035 in Ashkenazy Jewish | (2 fam)  1/1;0/1  1/1;1/1 | NF | (42, 43) | VUS |
| c.1864A>G/p.(Met622Val) | 194262 | 4 | 1.499 in Ashkenazy Jewish (2 Hmz) | (2 fam)  1/3;0/1  1/1;0/1 | 0,003  (1/286) | (42, 43) | Benign |
| c.-118A>C | 336058 | 3  1^§^ | NF | NA | NF | (8) | VUS |
| c.1890delT/p.(Phe630Leufs*8) | 1684420 | 3 | 0.005791 in Latino/Admixed American | (3 fam)  1/1;0/0  1/5;1/1  1/1;0/0 | NF | (41)  (sitosterolemia) | Likely pathogenic |
| c.593G>A/p.(Arg198Gln) | 284636 | 1  1^§^ | 0.2248 in European (non-Finnish) | NA | NF | (42, 43, 45) | VUS |
| c.80G>C/p.(Gly27Ala) | 283529 | 2 | 0.5532 in European (non-Finnish) (4 Hmz) | (1 fam)  0/1;0/0 | NF | (8, 42, 43) | Benign |
| c.1252G>A/p.(Asp418Asn) | 1761082 | 1  1^§^ | 0.0008790 in European (non-Finnish) | (1 fam)  0/0;1/2 | NF | No | VUS |
| c.862G>C/p.(Gly288Arg) | CA1636486 | 2 | 0.00008674 in Latino/Admixed American | (2 fam)  0/0;1/1  1/1;0/0 | NF | No | VUS |
| c.1228A>C/p.(Asn410His) | 785739 | 1 | 1.598 in African/African American (1 Hmz) | NA | NF | (43) | Benign |
| c.1888T>A/p.(Phe630Ile) | CA1636128 | 1^§^ | 0.001767 in European (non-Finnish) | NA | NF | No | VUS |
| c.757dup/p.(Arg253Profs*3) | CA2580612054 | 1 | NF | 3/3;0/0 | NF | No | Likely pathogenic |
| c.1550C>G/p.(Thr517Ser) | 779657 | 1^‡,†^ | 3.664 in African/African-American (19 Hmz) | NA | NF | (8, 43) | Benign |
| c.403_404dup/p.(Ser135Argfs*6) | CA2580612055 | 1 | NF | NA | NF | No | Likely pathogenic |
| c.139G>T/p.(Val47Phe) | 895028 | 1^‡,†^ | 0.9704 in African/African-American | NA | NF | (8, 43) | Benign |
| c.325T>C/p.(Phe109Leu) | 593676 | 1^†^ | 0.3655 in African/African American | 1/1;0/0 | NF | No | Likely benign |
| ***ABCG8*** |  | **N=23** |  |  |  |  |  |
| c.1974C>G/p.(Tyr658*) | 4969 | 3 | 0.02769 in other population | (1 fam)  1/1;0/0 | NF | (39, 40)  (sitosterolemia) | Likely pathogenic |
| c.644G>A/p.(Gly215Glu) | CA46441330 | 3 | NF | (1 fam)  1/1;0/0 | NF | No | VUS |
| c.1963A>G/p.(Met655Val) | 779658 | 2  1^†^ | 1.799 in African/African American (6 Hmz) | (2 fam)  1/1;0/0  1/1;0/0 | NF | No | Benign |
| c.1476T>A/p.(Tyr492*) | 1441157 | 2 | 0.008673 in Latino/Admixed American | (2 fam)  1/1;0;0  1/1;0;0 | NF | (38) (sitosterolemia) | Pathogenic |
| c.1201A>T/p.(Thr401Ser) | 198901 | 2 | 0.2278 in European (non-Finnish) | NA | NF | No | VUS |
| c.1177C>T/p.(Pro393Ser) | CA346669149 | 1^§^ | NF | NA | NF | No | VUS |
| c.462C>A/p.(His154Gln) | 1052851 | 1 | 0.01148 in African/African American (8710 alleles) | 1/1;0/0 | NF | No | VUS |
| c.1608G>A/p.(Trp536*) | 499930 | 1^§^ | 0.01129 in Latino/Admixed American | NA | NF | (39, 40)  (sitosterolemia) | Pathogenic |
| c.1688G>A/p.(Ser563Asn) | CA346670594 | 1 | NF | NA | NF | No | VUS |
| c.628G>A/p.(Val210Met) | 336070 | 1 | 4.754 in African/Admixed American (25 Hmz) | NA | NF | (43) | Benign |
| c.1106A>T/p.(Asp369Val) | 290382 | 1 | 0.006533 in South Asian | 0/0;1/1 | NF | No | VUS |
| c.712G>A/p.(Glu238Lys) | 291264 | 1 | 0.1568 in European (non-Finnish) | NA | NF | (8, 43) | VUS |
| c.898A>G/p.(Met300Val) | 1765338 | 1^†^ | 0.005783 in Latino/Admixed American | NA | NF | No | VUS |
| c.434G>C/p.(Arg145Thr) | CA1637015 | 1 | 0.002891 in Latino/Admixed American | NA | NF | No | VUS |
| c.1128-5C>T | 1555420 | 1 | 0.001764 in European (non-Finnish) | NA | NF | No | VUS |
| ***APOE*** |  | **N=4** |  |  |  |  |  |
| c.636_645del/p.(Val213Trpfs*35) | CA2580612068 | 1 | NF | 3/3;0/0 | NF | No | Pathogenic |
| c.262G>C/p.(Glu88Gln) | CA406303671 | 1 | 0.005791 in Latino/Admixed American | NA | NF | No | VUS |
| c.805C>G/p.(Arg269Gly) | 478884 | 1 | 0.09527 in other population | NA | NF | (10) | VUS |
| c.98G>A/p.(Arg33His) | CA406302480 | 1 | 0.001772 in European (non-Finnish) | NA | NF | No | VUS |
| ***LIPA*** |  | **N=1** |  |  |  |  |  |
| c.616G>A/p.(Val206Ile) | 301579 | 1 | 0.4446 in Ashkenazi Jewish | 1/1;0/0 | NF | No | VUS |

Htz, heterozygous; Hmz, homozygous; PopMAF: Population maximum allele frequency; Co-segregation is defined as carriers/affected; carriers/unaffected; NA: not applicable; NF: not found.

**^*^** Panel with 143 normolipidemic individuals (286 alleles)

^#^ Variants classified for the corresponding FH phenocopy: *ABCG5* and *ABCG8* for sitosterolemia, *APOE* for dysbetalipoproteinemia, *LIPA* for lysosomal acid lipase deficiency.

^§^ Individual carries two variants in same gene (phase unknown)

^‡^ Individual carries two variants in same gene (in cis)

^†^ Individual carries one variant in each gene (*ABCG5* and *ABCG8*)

**Supplemental Table S3**. List of pathogenic and likely pathogenic variants identified in FH-positive individuals of the Portuguese FH study cohort. For each variant are presented the LDLR activity (if appropriate functional studies were performed), the ACMG classification and criteria codes applied.

| **Gene** | **cDNA** | **Protein** | **ClinVar ID** | **Protein Activity** | **ACMG classification** | **Criteria codes applied** |
| --- | --- | --- | --- | --- | --- | --- |
| *LDLR* | c.-135C>G | p.(?) | 17890 | 5-15% LDLR activity | Pathogenic | PS4, PP1_Strong, PM2, PS3_Moderate, PP4 |
| *LDLR* | c.-57_67+56del (Ex1del) | p.(?) | 265885 | NP | Pathogenic | PVS1, PM2, PP4 |
| *LDLR* | c.1A>C | p.(Met1Leu) | 237735 | 60% cell surface LDLR, 59% binding and 66% uptake. 5% luciferase construct activity | Pathogenic | PS3, PP1_Moderate, PM2, PVS1_Moderate, PP4, PS4_Supporting |
| *LDLR* | c.77_78del | p.(Arg26Metfs*25) | 251008 | NP | Pathogenic | PVS1, PM2, PP4 |
| *LDLR* | c.190+1G>A | p.(?) | 251048 | NP | Likely pathogenic | PVS1_Strong, PM2, PS4_Supporting, PP4 |
| *LDLR* | c.190+2_190+3dup | p.Leu64Cysfs*143 | 251050 | retention of 2 nucleotides of intron 2 (p.Leu64Cysfs*143) | Likely pathogenic | PP1_Moderate, PM2, PS3_Supporting, PS4_Supporting, PP4 |
| *LDLR* | c.236dup | p.(Asn80Glnfs*50) | 251086 | NP | Pathogenic | PVS1, PM2, PP4, PP1_moderate |
| *LDLR* | c.241C>T | p.(Arg81Cys) | 183083 | LDL-uptake 68% | Likely pathogenic | PM2, PP4, PP3, PS4_supporting, PS3_supporting |
| *LDLR* | c.261G>A | p.(Trp87*) | 251100 | 5-10% LDLR expression and 5-10% LDLR activity | Pathogenic | PVS1, PS4, PM2, PS3_Supporting, PP4 |
| *LDLR* | c.265T>C | p.(Cys89Arg) | 251102 | NP | Likely pathogenic | PM1, PM2, PM3, PP1, PP3, PP4, PS4_Supporting |
| *LDLR* | c.301G>A | p.(Glu101Lys) | 161266 | 15-30% LDLR activity | Pathogenic | PS4, PP1_Strong, PM2, PS3_Moderate, PM3, PP3, PP4 |
| *LDLR* | c.310_313del | p.(Cys104Profs*101) | 251128 | NP | Pathogenic | PVS1, PM2, PM1, PP4, PP1 |
| *LDLR* | c.313+1G>A | p.(?) | 3736 | skipping of exon 3 or inclusion of intron 3; 12% LDL-LDLR uptake in Hmz | Pathogenic | PVS1_Strong, PS4, PP1_Strong, PM2, PS3_Moderate, PP4 |
| *LDLR* | c.313+1G>C | p.(?) | 228358 | NP | Pathogenic | PS4, PVS1_Strong, PM2, PP4 |
| *LDLR* | c.313+1G>T | p.(?) | 251134 | skipping of exon 3 (p.Leu64_Pro105delinsSer) | Likely pathogenic | PVS1_Strong, PM2, PP4, PS3_Supporting, PS4_Supporting |
| *LDLR* | c.313+6T>C | p.(?) | 251138 | skipping of exon 3 (p.Leu64_Pro105delinsSer) | Likely pathogenic | PM2, PM3, PS3_Supporting, PP4 |
| *LDLR* | c.314-1G>A | p.Pro106_Ala232del | 251148 | skipping of exon 4 (p.Pro106_Ala232del), 41% LDLR activity. | Pathogenic | PVS1_Strong, PM2, PS3_Moderate, PP4, PS4_Supporting |
| *LDLR* | c.324_325delinsTC | p.(Cys109Arg) | 251154 | NP | Likely pathogenic | PM1, PM2, PP1, PP4, PS4_Supporting |
| *LDLR* | c.326G>T | p.(Cys109Phe) | 251157 | Normal cell surface LDLR (100%), 43% LDL-LDLR binding and 34% uptake | Pathogenic | PS3, PP1_moderate, PM1, PM2, PP3, PP4 |
| *LDLR* | c.369_370del | p.(Arg124Alafs*5) | 251182 | NP | Pathogenic | PVS1, PM2, PP4 |
| *LDLR* | c.369_393del | p.(Arg124Glyfs*74) | 251183 | NP | Pathogenic | PVS1, PM2, PP1, PP4, PS4_Supporting |
| *LDLR* | c.418G>A | p.(Glu140Lys) | 251213 | >100% LDLR expression and 37-42% LDLR activity | Pathogenic | PM1, PM2, PP1_Strong, PP3, PP4, PS3_Supporting, PS4_Supporting |
| *LDLR* | c.427T>C | p.(Cys143Arg) | 251219 | Normal cell surface LDLR (94%), 43% LDL-LDLR binding and 34% uptake | Pathogenic | PS3, PM1, PM2, PP1, PP3, PP4 |
| *LDLR* | c.473C>G | p.(Ser158Cys) | 251245 | 78-86% LDLR expression and 48-49% LDLR activity | Likely pathogenic | PM1, PM2, PP4, PS3_Supporting, BP4 |
| *LDLR* | c.479G>T | p.(Cys160Phe) | 441187 | NP | Likely pathogenic | PM2, PM1, PP3, PP4 |
| *LDLR* | c.502G>C | p.(Asp168His) | 251258 | <2% LDLR activity | Pathogenic | PS4, PM1, PM2, PS3_Moderate, PP3, PP4 |
| *LDLR* | c.530C>T | p.(Ser177Leu) | 3686 | 65% cell surface LDLR, 10% binding and <2% uptake | Pathogenic | PS3, PS4, PP1_Strong, PM1, PM2, PM3, PP3, PP4, BS4 |
| *LDLR* | c.551G>A | p.(Cys184Tyr) | 3739 | Normal cell surface LDLR (100%), 4% LDL-LDLR binding and 18% uptake | Pathogenic | PS3, PS4, PP1_Strong, PM2, PM1, PP3, PP4 |
| *LDLR* | c.589T>C | p.(Cys197Arg) | 183091 | 100% LDLR expression and 27-37% LDLR activity | Likely pathogenic | PM2, PM1, PP1_Moderate, PP3, PS3_Supporting, PS4_Supporting, PP4 |
| *LDLR* | c.590G>T | p.(Cys197Phe) | 251309 | 100% LDLR expression and 31-43% LDLR activity | Likely pathogenic | PM1, PM2, PP1_moderate, PP3, PP4, (PS3_Supporting) |
| *LDLR* | c.618_638del | p.(Gly207_Ser213del) | 265899 | Normal cell surface LDLR (96%), 10% LDL-LDLR binding and 7% uptake | Pathogenic | PS3, PS4, PP1_Strong, PM2, PM4, PP4 |
| *LDLR* | c.631C>G | p.(His211Asp) | 251334 | Normal cell surface LDLR (100%), 38% binding and 50% uptake | Pathogenic | PS3, PM1, PM2, PP1, PP3, PP4 |
| *LDLR* | c.661G>T | p.(Asp221Tyr) | 251356 | Normal cell surface LDLR (106%), 5% LDL-LDLR binding and 8% uptake | Pathogenic | PS3, PP1_Strong, PM1, PM2, PS4_Moderate, PP3, PP4 |
| *LDLR* | c.662A>G | p.(Asp221Gly) | 183092 | 8% LDLR activity | Pathogenic | PS4, PP1_Strong, PM1, PM2, PM3, PS3_Moderate, PP3, PP4, BS4 |
| *LDLR* | c.665G>A | p.(Cys222Tyr) | 183093 | NP | Likely pathogenic | PM2, PM1, PP3, PP4, PS4_Supporting |
| *LDLR* | c.666C>G | p.(Cys222Trp) | 251365 | >100% LDLR expression and 31-43% LDLR activity | Likely pathogenic | PM1, PM2, PP3, PP4, PS3_Supporting |
| *LDLR* | c.670G>A | p.(Asp224Asn) | 3706 | <2% LDLR activity | Pathogenic | PS4, PP1_Strong, PM2, PM1, PM3, PS3_Moderate, PP3, PP4 |
| *LDLR* | c.670G>A | p.[(Asp224Asn)];[(Glu716Lys)] | 3706 | <2% LDLR activity | Pathogenic | PS4, PP1_Strong, PM2, PM1, PM3, PS3_Moderate, PP3, PP4 |
| *LDLR* | c.682dup | p.(Glu228Glyfs*12) | CA1139532995 | NP | Pathogenic | PVS1, PM2, PP4, PP1, PS4_Supporting |
| *LDLR* | c.682G>A | p.(Glu228Lys) | 3691 | <2% LDLR activity | Pathogenic | PS3, PS4, PM1, PM2, PP1_Strong, PP3, PP4 |
| *LDLR* | c.691T>G | p.(Cys231Gly) | 251397 | 94-100% LDLR expression and 29-35% LDLR activity | Likely pathogenic | PM1, PM2, PP3, PP4, PS3_Supporting, PS4_Supporting |
| *LDLR* | c.693C>G | p.(Cys231Trp) | 251400 | NP | Likely pathogenic | PM2, PM1, PP3, PP4, PS4_Supporting |
| *LDLR* | c.799G>A | p.(Glu267Lys) | 251459 | Normal cell surface LDLR (95%), 63% LDL-LDLR binding and 61% uptake | Likely pathogenic | PS3, PM2, PP3, PP4 |
| *LDLR* | c.818-2A>G | p.(?) | 251471 | Retention of 10 nt of intron5 (p.Val273Glyfs*31) | Pathogenic | PVS1, PM2, PP1_Moderate, PS3_Supporting, PS4_Supporting, PP4 |
| *LDLR* | c.862G>A | p.(Glu288Lys) | 161268 | Normal cell surface LDLR (108%), 10% binding and 7% uptake | Pathogenic | PP1_Strong, PM2, PS4_Moderate, PS3_Supporting, PP3, PP4 |
| *LDLR* | c.939C>A | p.(Cys313*) | 251539 | <2% LDLR activity | Pathogenic | PVS1, PS3_moderate, PM2, PM1, PP1 |
| *LDLR* | c.941-2A>C | p.(?) | 251553 | NP | Likely pathogenic | PVS1_Strong, PM2, PP4, PS4_Supporting |
| *LDLR* | c.1017dup | p.(Cys340Valfs*18) | 265901 | NP | Pathogenic | PVS1, PM2, PP1, PP4 |
| *LDLR* | c.1027G>A | p.(Gly343Ser) | 183106 | >100% LDLR expression and 64-69% LDLR activity | Pathogenic | PS3, PS4, PP1_Strong, PM2, PM3, PM5, PP3, PP4, BS4 |
| *LDLR* | c.1033C>T | p.(Gln345*) | 226341 | NP | Pathogenic | PVS1, PM2, PP1, PP4 |
| *LDLR* | c.1048C>T | p.(Arg350*) | 226342 | NP | Pathogenic | PVS1, PS4, PP1_Strong, PM2, PP4 |
| *LDLR* | c.1054T>A | p.(Cys352Ser) | 251617 | Normal cell surface LDLR (93%), 47% LDL-LDLR binding and 45% uptake | Pathogenic | PS3, PM2, PM1, PP3, PP4 |
| *LDLR* | c.1060+1G>A | p.(?) | 251623 | skipping of exon 7 (p.Gly314_Glu353del) | Pathogenic | PVS1_Strong, PS4, PP1_Strong, PM2, PS3_Supporting, PP4 |
| *LDLR* | c.1085del | p.(Asp362Alafs*8) | 251655 | NP | Pathogenic | PVS1, PM2, PP4 |
| *LDLR* | c.1165del | p.(Thr389Argfs*24) | CA1139532993 | NP | Pathogenic | PVS1, PM2, PP4 |
| *LDLR* | c.1176C>A | p.(Cys392*) | 251699 | NP | Pathogenic | PVS1, PP1_Strong, PM2, PM3, PS4_Moderate, PP4 |
| *LDLR* | c.1178del | p.(Lys393Argfs*20) | 251698 | NP | Pathogenic | PVS1, PM2, PM3, PP4 |
| *LDLR* | c.1216C>T | p.(Arg406Trp) | 226351 | 60-65% LDLR cell surface, LDL-LDLR binding and internalization; reduced mature protein | Pathogenic | PS3, PS4, PP1_Strong, PM2, PP3, PP4 |
| *LDLR* | c.1222G>A | p.(Glu408Lys) | 36453 | <2% LDLR activity | Likely pathogenic | PM2, PS3_moderate, PS4_Moderate, PP3, PP4, PP1 |
| *LDLR* | c.1247G>A | p.(Arg416Gln) | 251752 | NP | Likely pathogenic | PM2, PM5, PP3, PP4, PS4_Supporting |
| *LDLR* | c.1285G>C | p.(Val429Leu) | 226353 | 15-30% LDLR activity; reduced mature protein | Pathogenic | PS3, PM2, PM5, PP3, PP4, PS4_Supporting |
| *LDLR* | c.1291G>A | p.(Ala431Thr) | 3695 | 22% LDLR expression, 20% LDL-LDLR binding and internalization | Pathogenic | PS3, PS4, PP1_Strong, PM2, PM3, PP3, PP4 |
| *LDLR* | c.1322T>C | p.(Ile441Thr) | 251783 | 7% cell surface LDLR, 5% LDL-LDLR binding, 10% LDL-LDLR internalization; no mature protein detected | Pathogenic | PS3, PP1_Strong, PM2, PS4_Moderate, PP3, PP4 |
| *LDLR* | c.1325A>G | p.(Tyr442Cys) | 265906 | 5-10% LDLR expression and 5-10% LDLR activity | Likely pathogenic | PM2, PP3, PP4, PS3_Supporting, PS4_Supporting (PM5 not assessed) |
| *LDLR* | c.1352T>C | p.(Ile451Thr) | 251801 | 7% cell surface LDLR, 11% binding and 4% uptake | Pathogenic | PS3, PM2, PP1, PP3, PP4, PS4_Supporting (PM5 not evaluated) |
| *LDLR* | c.1359-5C>G | p.(?) | 251808 | Retention of intron 9 (p.Ser453Argfs*2) | Likely pathogenic | PP1_moderate, PM2, PP4, PS3_supporting, PS4_supporting |
| *LDLR* | c.1374_1375del | p.(Arg458Serfs*8) | 251818 | NP | Pathogenic | PVS1, PM2, PP4, PP1 |
| *LDLR* | c.1432G>A | p.(Gly478Arg) | 161277 | 33-43% LDLR expression and 66-68% LDLR activity | Pathogenic | PP1_Strong, PS4, PM2, PP3, PP4 (PS3_Supporting) |
| *LDLR* | c.1433G>A | p.(Gly478Glu) | 440642 | NP | Likely pathogenic | PM2, PM5, PP3, PP4 |
| *LDLR* | c.1455C>G | p.(His485Gln) | 251851 | 54% cell surface LDLR, 50% LDL-LDLR binding and 61% uptake | Likely pathogenic | PS3, PM2, PP1, PP4 |
| *LDLR* | c.1463T>C | p.(Ile488Thr) | 3710 | 20-39% LDLR expression and 45-63% LDLR activity | Likely pathogenic | PM2, PP1, PP3, PP4, PS3_Supporting, PS4_Supporting |
| *LDLR* | c.1468T>C | p.(Trp490Arg) | 180402 | 5-15% LDLR activity; reduced mature protein | Pathogenic | PM2, PP3, PS3, PP4, PS4_Moderate, PP1_Moderate |
| *LDLR* | c.1474G>A | p.(Asp492Asn) | 161285 | ~60% cell surface LDLR, LDL-LDLR binding and uptake | Pathogenic | PS3, PS4, PM2, PP3, PP4 |
| *LDLR* | c.1499T>C | p.(Val500Ala) | 265903 | 46% cell surface LDLR, 48% LDL-LDLR binding and 57% uptake | Likely pathogenic | PS3, PM2, PP1, PP3, PP4 |
| *LDLR* | c.1585G>C | p.(Gly529Arg) | 183122 | 69% LDL-LDLR binding | Pathogenic | PM2, PP3, PS4_Supporting, PP4, PS3, PM3 |
| *LDLR* | c.1586+2T>A | p.(?) | 251907 | NP | Likely pathogenic | PVS1_Strong, PM2, PP4 |
| *LDLR* | c.1598G>A | p.(Trp533*) | 251926 | NP | Pathogenic | PVS1, PM2, PP4, PS4_Supporting |
| *LDLR* | c.1599G>A | p.(Trp533*) | 251927 | NP | Pathogenic | PVS1, PM2, PP4, PS4_Supporting, PP1_Supporting |
| *LDLR* | c.1618_1620del | p.(Ala540del) | 251937 | NP | Likely pathogenic | PM2, PM4, PP4, PP1 |
| *LDLR* | c.1618G>A | p.(Ala540Thr) | 226363 | cell surface LDLR 40-50% LDLR | Pathogenic | PS4, PP1_Strong, PM2, PM3, PP3, PP4, PS3_Supporting |
| *LDLR* | c.1633G>T | p.(Gly545Trp) | 251944 | 5-10% LDLR cell surface, LDL-LDLR binding and internalization; no mature protein detected | Pathogenic | PS3, PS4, PP1_Strong, PM2, PP4 |
| *LDLR* | c.1646G>A | p.(Gly549Asp) | 3698 | <2% LDLR activity | Pathogenic | PS4, PM2, PS3_Moderate, PP3, PP4, PP1 (some case data codes not evaluated) |
| *LDLR* | c.1659_1661delinsATACTTTCA | p.(Tyr553*) | 251958 | NP | Pathogenic | PVS1, PM2, PP4 |
| *LDLR* | c.1690A>C | p.(Asn564His) | 226365 | NP | Likely pathogenic | PS4, PM2, PP3, PP4 |
| *LDLR* | c.1731G>A | p.(Trp577*) | 226370 | <2% LDLR activity | Pathogenic | PVS1, PM2, PS3_Moderate, PP4 |
| *LDLR* | c.1756del | p.(Ser586Glnfs*79) | 252019 | NP | Pathogenic | PVS1, PM2, PP1, PP4 |
| *LDLR* | c.1775G>A | p.(Gly592Glu) | 226397 | 51% cell surface LDLR, 35% LDL-LDLR binding and 40% uptake | Pathogenic | PS4, PP1_Strong, PM2, PS3_Moderate, PP3, PP4 |
| *LDLR* | c.1801G>C | p.(Asp601His) | 252038 | NP | Likely pathogenic | PM2, PM5, PP4, PS4_Supporting |
| *LDLR* | c.1802A>T | p.(Asp601Val) | 252040 | 4% cell surface LDLR, 9% LDL-LDLR binding and 10% uptake | Pathogenic | PP1_Strong, PS3, PM2, PP4 |
| *LDLR* | c.1823C>A | p.(Pro608His) | 2636939 | <5% LDLR expression and LDLR activity | Likely pathogenic | PS3, PM2, PP3, PP4 |
| *LDLR* | c.1840T>A | p.(Phe614Ile) | 252061 | ~50% cell surface LDLR, LDL-LDLR binding and uptake | Likely pathogenic | PS3, PM2, PP1, PP3, PP4 |
| *LDLR* | c.1845+1del | p.(?) | 252066 | NP | Likely pathogenic | PVS1_Strong, PM2, PS4_Supporting, PP4 |
| *LDLR* | c.1846-1G>A | p.(?) | 252079 | <2% LDLR activity; alternative splicing | Pathogenic | PVS1, PM2, PS3_moderate, PP4, PS4_supporting |
| *LDLR* | c.1876G>A | p.(Glu626Lys) | 183127 | Normal cell surface LDLR (98%), 84% LDL-LDLR binding and 58% LDL-LDLR uptake | Pathogenic | PP1_Strong, PS3 |
| *LDLR* | c.1886del | p.(Phe629Serfs*36) | 252104 | NP | Pathogenic | PVS1, PM2, PP4, PS4_Supporting |
| *LDLR* | c.1897C>T | p.(Arg633Cys) | 226379 | NP | Likely pathogenic | PM2, PP1_Moderate, PS4_Moderate, PP3, PP4 |
| *LDLR* | c.1934dup | p.(Asn645Lysfs*24) | 252116 | NP | Pathogenic | PVS1, PP1_Strong, PM2, PP4, PS4_Supporting |
| *LDLR* | c.1936del | p.(Leu646Tyrfs*19) | 252119 | NP | Pathogenic | PVS1, PM2, PP4 |
| *LDLR* | c.1942T>C | p.(Ser648Pro) | 252120 | 30-50% LDLR activity; reduced mature protein | Likely pathogenic | PS3, PM2, PP4 |
| *LDLR* | c.1959del | p.(Leu654Serfs*11) | CA2573320554 | NP | Pathogenic | PVS1, PM2, PP4 |
| *LDLR* | c.1999T>C | p.(Cys667Arg) | 252163 | 5-10% LDLR expression and 5-10% LDLR activity | Likely pathogenic | PM1, PM2, PP3, PP4, PS3_Supporting, PS4_Supporting |
| *LDLR* | c.2054C>T | p.(Pro685Leu) | 3702 | 20-25% LDLR activity | Pathogenic | PS4, PP1_Strong, PM2, PM3, PM5, PS3_Moderate, PP3, PP4 |
| *LDLR* | c.2056C>T | p.(Gln686*) | 252199 | NP | Pathogenic | PVS1, PM2, PP1_Moderate, PP4, PS4_Supporting |
| *LDLR* | c.2077_2078del | p.(Lys693Valfs*23) | 252207 | NP | Pathogenic | PVS1, PP1_Strong, PM2, PS4_Moderate, PP4 |
| *LDLR* | c.2093G>T | p.(Cys698Phe) | 252216 | 5-10% LDLR cell surface, LDL-LDLR binding and internalization; no mature protein detected | Pathogenic | PS3, PM2, PM1, PP3, PP4 |
| *LDLR* | c.2140+1G>A | p.(?) | 3744 | 50% LDLR activity | Pathogenic | PVS1, PP1_Strong, PM2, PS4_Moderate, PS3_Supporting, PP4 |
| *LDLR* | c.2214del | p.(Gln739Serfs*26) | CA1139768693 | NP | Pathogenic | PVS1, PM2, PP4 |
| *LDLR* | c.2385del | p.(Ile796Serfs*133) | 252297 | NP | Pathogenic | PVS1, PM2, PP4 |
| *LDLR* | c.2389G>A | p.Ala771Valfs*17 | 226393 | skipping of exon 16 | Pathogenic | PP1_Strong, PM2, PP4, PS3_Supporting, PS4_Moderate |
| *LDLR* | c.2389G>T | p.Ala771Valfs*17 | 252298 | skipping of exon 16 | Likely pathogenic | PM2, PP1, PP4, PS3_Supporting, PS4_Supporting |
| *LDLR* | c.2397_2411del | p.(Val800_Leu804del) | CA1139654948 | NP | Likely pathogenic | PM2, PM4, PS4_Supporting, PP1, PP4 |
| *LDLR* | c.2399_2403delinsGGGT | p.(Val800Glyfs*129) | 252322 | 7% LDL-LDLR binding, uptake and cell surface LDLR | Pathogenic | PVS1, PS3, PM2, PP4, PS4_Supporting |
| *LDLR* | c.2416dup | p.(Val806Glyfs*11) | 252330 | NP | Pathogenic | PVS1, PP1_Strong, PS4, PM2, PP4 |
| *LDLR* | c.2547+1G>A | p.(?) | 252352 | skipping of exon 17 (p.Val797Glufs*9) | Pathogenic | PVS1, PM2, PS3_Supporting, PS4_Supporting, PP4, PP1 |
| *LDLR* | c.2548-3_2557del | p.(?) | CA2277442043 | NP | Pathogenic | PVS1, PM2, PP4 |
| *LDLR* | c.[1-?_190+?del; 1061-?_1845+?del] (Pr_EX2del + EX8_12del) | p.(?) | cannot have CA | NP | Pathogenic | PVS1, PS4, PP1_Strong, PM2, PP4 |
| *LDLR* | c.(67+1_68-1)_(313+1_314-1)del (EX2_3del) | p.(?) | 3710 | 30% LDLR activity | Pathogenic | PVS1_Strong, PM2, PS3_Moderate, PP1, PP4, PS4_Supporting |
| *LDLR* | c.(1060+1_1061-1)_(1845+1_1846-1)del (EX8_12del) | p.(?) | 265901 | NP | Pathogenic | PVS1, PP1_Moderate, PM2, PP4 |
| *LDLR* | c.(1586+1_1587-1)_(1845+1_1846-1)del (EX11_12del) | p.Phe530Thrfs*49 | 226397 | skipping of exons 11 and 12 (p.Phe530Thrfs*49); 40% LDLR activity | Pathogenic | PVS1,PS3_Moderate, PM2, PP4 |
| *LDLR* | c.1846-?_2311+?dup (EX13_15dup) | p.(?) | 252077 | NP | Likely pathogenic | PVS1_Strong, PM2, PP1, PP4 |
| *LDLR* | c.2312-?_2389+?del (EX16del) | p.(Ala771_Ile796del) | 431546 | NP | Pathogenic | PVS1_Strong, PM2, PS3_Moderate, PP4, PS4_Supporting, PP1 |
| *LDLR* | c.(2311+1_2312-1)_(*_?)del (EX16_18del) | p.(?) | 265906 | Normal LDL-LDLR binding, 2-5% LDL-LDLR uptake, <2% LDLR degradation | Pathogenic | PVS1, PM2, PS3_Moderate, PP1, PP4, PS4_Supporting |
| *APOB* | c.13480_13482del | p.(Gln4494del) | 265896 | 40% proliferation; 50-60% binding and internalization | Pathogenic | PS3, PM1, PM4, PM8, PP4, BS4 |
| *APOB* | c.11477C>T | p.(Thr3826Met) | 237735 | 40% proliferation; 50-60% binding and internalization | Likely pathogenic | PS3, PM8, PP4 |
| *APOB* | c.10580G>A | p.(Arg3527Gln) | 17890 | 40-50 % cells proliferation / 40-50% LDL binding and uptake | Pathogenic | PS3, PM5, PM8, PM9, PP4 |
| *APOB* | c.3491G>C | p.(Arg1164Thr) | 265885 | 40% proliferation; 50-60% binding and internalization | Likely pathogenic | PS3, PM8, PP4, BS4 |
| *PCSK9* | c.185C>A | p.(Ala62Asp) | 265918 | decrease of 46% on cell surface expression and decrease of 35% in LDL uptake. | Likely pathogenic | PS3, PM2, PP4 |
| *PCSK9* | c.1120G>C | p.(Asp374His) | 265939 | 35% LDLR activity (HEK: normal autocatalitic activity and PCSK9 secretion; HEK + Lymphocytes: 20-30% LDLR amount (75% reduction) | Pathogenic | PS3, PM2, PM5, PP1, PP4 |
| *PCSK9* | c.1399C>G | p.(Pro467Ala) | 265944 | decrease 56% cell surface expression and decrease of 35% in LDL uptake. | VUS | PS3, PP4 |

*LDLR* variants were classified according to the American College of Medical Genetics and Genomics (ACMG) guidelines (25) and the Clinical Genome Resource (ClinGen) Familial Hypercholesterolemia Variant Curation Expert Panel (FH VCEP) consensus guidelines for *LDLR* variant classification (30). *APOB* and *PCSK9* variants were classified according to the ACMG guidelines (25),~~,~~ using specific published adaptations for these genes (31). NP: appropriate functional studies not performed.

**Supplemental Table S4.** Demographic, clinical characteristics and lipid profile of the Portuguese FH cohort.

|  | Pediatric cohort  (N=408) | | | | Adult cohort  (N= 557) | | | |
| --- | --- | --- | --- | --- | --- | --- | --- | --- |
|  | Total | FH-positive | FH-negative | *P* value | total | FH-positive | FH-negative | *P* value |
| N (%) |  | 194 (47.5) | 214 (52.5) |  |  | 214 (38.4) | 343 (61.6) |  |
| Male  N (%) | 184 (45.1) | 98 (50.5) | 86 (40.2) | 0.058 | 241 (43.3) | 86 (40.2) | 155 (45.2) | 0.292 |
| Age (years) | 10.2±3.7 | 10.2±3.8 | 10.2±3.6 | 0.796 | 45.0±13.9 | 43.3±14.9 | 46.0±13.1 | 0.014 |
| BMI (kg/m^2^) | 19.6±4.0 | 19.2±4.0 | 20.0±4.2 | 0.045 | 26.0±4.4 | 26.1±4.7 | 26.0±4.2 | 0.587 |
| Presence of CAD  N (%) | 0 (0) |  |  |  | 115 (20.6) | 44 (20.6) | 71 (20.7) | 1 |
| Age 1^st^ event (years) | 0 (0) |  |  |  | 45.8±10.5 | 45.6±11.5 | 45.9±9.5 | 0.909 |
| Presence of pCAD  N (%) | 0 (0) |  |  |  | 98 (17.6) | 36 (16.8) | 62 (18.1) | 0.819 |
| Age 1^st^ event (years) | 0 (0) |  |  |  | 43.9±9.2 | 42.8±9.8 | 44.3±8.2 | 0.431 |
| Tendon xanthoma  N (%) | 1 (0.2) | 1 (0.5) | 0 (0) |  | 28 (5) | 21 (9.8) | 7 (2) | <0.001* |
| LLT  N (%) | 106 (26) | 64 (33) | 42 (19.6) | 0.019* | 427 (76.7) | 166 (77.6) | 261 (76.1) | 0.090 |
|  |  |  |  |  |  |  |  |  |
| *N* | *379* | *181* | *198* |  | *499* | *191* | *308* |  |
| TC† (mg/dL) | 260.5±53.8 | 279.5±47.1 | 238.0±41.6 | 0.000* | 305.5±81.3 | 334.5±91.3 | 284.5±64.8 | <0.001* |
| LDL-c† (mg/dL) | 191.3±57.4 | 217.1±49.7 | 162.1±38.8 | 0.000* | 232.4±86.8 | 268.2±96.6 | 206.8±66.8 | <0.001* |
| HDL-c† (mg/dL) | 55.3±15.3 | 51.1±12.4 | 59.2±16.9 | <0.001* | 55.3±17.0 | 54.7±14.3 | 55.9±17.1 | 0.253 |
| TG† (mg/dL) | 87.5±46.2 | 77.3±35.1 | 95.1±49.7 | <0.001* | 136.8±73.7 | 121.4±79.4 | 146.6±69.4 | <0.001* |
| apoB† (mg/dL) | 116.9±33.7 | 127.3±28.3 | 104.5±26.3 | <0.001* | 131.2±46.9 | 149.7±52.4 | 118.3±38.4 | <0.001* |
| apoA1† (mg/dL) | 143.9±29.9 | 133.4±22.2 | 153.6±30.7 | <0.001* | 154.8±35.1 | 147.6±34.8 | 160,2±34.7 | <0.001* |
| apoB/apoA1 Ratio† (mg/dL) | 0.85±0.33 | 0.98±0.29 | 0.71±0.26 | 0.000* | 0.90±0.44 | 1.08±0.52 | 0.78±0.34 | <0.001* |
| Lp(a)† (mg/dL) | 49.9±58.1 | 40.6±42.8 | 57.2±64.9 | 0.084 | 54.1±57.7 | 54.2±55.3 | 53.7±60.2 | 0.347 |
| Hyper Lp(a)  N (%) | 129 (34) | 46 (25.4) | 83 (41.9) | <0.001* | 183 (36.7) | 73 (38.2) | 110 (35.7) | 0.319 |
|  |  |  |  |  |  |  |  |  |
| *N* | *84* | *36* | *48* |  | *118* | *35* | *83* |  |
| *LPA* risk score  N (%) | 56 (66.7) | 22 (61.1) | 34 (70.8) | 0.362 | 75 (63.6) | 25 (71.4) | 50 (60.2) | 0.298 |
|  |  |  |  |  |  |  |  |  |
| N | *307* | *137* | *170* |  | *401* | *146* | *255* |  |
| PRS | 0.71±0.19 | 0.67±0.20 | 0.74±0.17 | 0.007* | 0.69±0.19 | 0.67±0.20 | 0.71±0.19 | 0.044* |
| High PRS  N (%) | 137 (44.6) | 53 (38.7) | 84 (49.4) | 0.065 | 161 (40.1) | 55 (37.7) | 106 (41.6) | 0.460 |

Data are expressed as mean ± standard deviation, unless otherwise noted.

Individuals with homozygous FH, VUS in *LDLR* and other monogenic causes are not included.

LLT, lipid lowering therapy; PRS, Polygenic risk score; CAD defined if any of the events occurred: angina, myocardial infraction, percutaneous transluminal coronary angioplasty (PTCA) or coronary artery bypass graft (CABG); pCAD indicates CAD <55 years old in males and <65 years old in females; Hyper Lp(a) defined if Lp(a) values >50mg/dl; *LPA* risk score defined for genotype score ≥1. High PRS was considered for genetic risk score ≥0.76 (>75th percentile).

†Lipid profile determined at INSA at the time of entry into the Portuguese FH Study. If untreated values were not available, 0.8 and 0.7 correction factors were applied to TC or LDL-c values, respectively, for individuals undertreatment (30).

**P*<0.05 FH-positive versus FH-negative.

**Supplemental Table S5.** Individuals carrying 2 variants in *ABCG5* and *ABCG8* genes.

| **Genes** | **Variants** | **% popMAF (GnomAD v2.1.1)** | **Beta-sitosterol (µmol/L)** |
| --- | --- | --- | --- |
| ***ABCG5* and *ABCG8*** | *ABCG5*: c.[139G>T;1550C>G]/p.[(Val47Phe);(Thr517Ser)]  *ABCG8*: c.898A>G/p.(Met300Val) | 0.9704 and 3.664 in African/African American 0.005783 in Latino/Admixed American | Not detected |
|  | *ABCG5*: c.325T>C/p.(Phe109Leu) *ABCG8*: c.1963A>G/p.(Met655Val) | 0.3655 in African/African American 1.799 in African/African American | 13.1 |
| ***ABCG5*** | c.[-118A>C](;)[1888T>A]/p.[(?)](;)[(Phe630Ile)] | NF 0.001767 in European (non-Finnish) | NP |
|  | c.[593G>A](;)[1252G>A]/p.[(Arg198Gln)](;)[(Asp418Asn)] | 0.2248 in European (non-Finnish) 0.0008790 in European (non-Finnish) | 6.5 |
| ***ABCG8*** | c.[1177C>T](;)[1608G>A]/p.[(Pro393Ser)](;)[(Trp536*)] | NF 0.01129 in Latino/Admixed American | 9.1 |

PopMAF: Population maximum allele frequency; NP: beta-sitosterol not determined.

**Supplemental Table S6**. LDL-c mean levels of the FH-positive and FH-negative subgroups.

|  | Pediatric cohort | | | Adult cohort | | |
| --- | --- | --- | --- | --- | --- | --- |
| Subgroups | N | LDL-c (mg/dL) | *P*-value | N | LDL-c (mg/dL) | *P*-value |
| FH-positive | 181 | 217.1±49.5 |  | 191 | 267.7±96.6 |  |
| FH-negative with variants in FH phenocopies genes | 16 | 168.6±21.6 | <0.001*  0.581^#^  0.553^§^  0.469^‡^  0.273^†^ | 34 | 215.5±84 | <0.001*  0.311^#^  0.467^§^  0.420^‡^  0.856^†^ |
| FH-negative with hyper-Lp(a) | 39 | 165.9±41.3 | <0.001*  0.955^§^  0.824^‡^  0.484^†^ | 61 | 203±64.5 | <0.001*  0.772^§^  0.873^‡^  0.244^†^ |
| FH-negative with polygenic | 38 | 163.1±42 | <0.001*  0.870^‡^  0.529^†^ | 50 | 204.7±58 | <0.001*  0.396^‡^  0.450^†^ |
| FH-negative with hyper-Lp(a) and polygenic | 41 | 161.9±35.5 | <0.001*  0.647^†^ | 43 | 206.9±82.3 | <0.001*  0.396^†^ |
| FH-negative with unknown causes | 64 | 157.8±40.5 | <0.001* | 120 | 211.2±64.7 | <0.001* |

Data are expressed as mean ± standard deviation.

* versus FH-positive. # versus FH-negative individuals with hyper-Lp(a). § versus FH-negative individuals with polygenic. ‡ versus FH-negative individuals with hyper-Lp(a) and polygenic. † versus FH-negative individuals with unknown causes.

**Supplemental Figure 1**. Flow diagram of the methodology used between 1999-2021 for the molecular study of index-cases of the Portuguese FH study.
